# Anterior Scleral Thickness and Other Dimensions in Nanophthalmos by Swept-Source Optical Coherence Tomography: A Comparative Study

**DOI:** 10.3390/jcm12175564

**Published:** 2023-08-26

**Authors:** José Ignacio Fernández-Vigo, Olivia Rodríguez-Quet, Elena Montolío-Marzo, Bárbara Burgos-Blasco, Bachar Kudsieh, Julio González-Martin-Moro, Julián García-Feijóo

**Affiliations:** 1 Department of Ophthalmology, Hospital Clínico San Carlos, Instituto de Investigación Sanitaria (IdISSC), 28040 Madrid, Spain; oliviaroq@gmail.com (O.R.-Q.); elenamontimar@gmail.com (E.M.-M.); bburgos171@hotmail.com (B.B.-B.); jgarciafeijoo@hotmail.com (J.G.-F.); 2 Department of Ophthalmology, Centro Internacional de Oftalmología Avanzada, 28010 Madrid, Spain; bacharkudsieh@gmail.com; 3 Department of Ophthalmology, Hospital Puerta de Hierro-Majadahonda, 28220 Madrid, Spain; 4 Department of Ophthalmology, Hospital Universitario del Henares, 28822 Madrid, Spain; juliogazpeitia@gmail.com; 5 Faculty of Medicine, Universidad Francisco de Vitoria, 28223 Madrid, Spain

**Keywords:** nanophthalmos, anterior scleral thickness, choroidal thickness, optical coherence tomography, anterior chamber depth

## Abstract

Purpose: The purpose of this study is to assess the ocular dimensions of the anterior and posterior segment, including the anterior scleral thickness (AST) in nanophthalmos compared to control eyes. Methods: A cross-sectional comparative study was carried out in two groups: 46 eyes of 28 patients with nanophthalmos, defined as axial length (AXL) < 20.5 mm, and 60 eyes of 30 controls paired by age and sex. The AST and ocular wall thickness (OWT) were measured by optical coherence tomography in the temporal and nasal quadrants at 1, 2, and 3 mm from the scleral spur. Also, the anterior chamber depth (ACD), white-to-white (WTW), lens thickness (LT), subfoveal choroidal thickness (SFCT), and retinal thickness (RT) were evaluated. Results: The mean AXL was 19.3 ± 1.5 mm in the nanophthalmos group and 23.9 ± 1.1 mm in the control group (*p* < 0.001). The OWT was thicker in all measurement points in nanophthalmos (*p* < 0.001). There were no differences in the AST measurements between groups, except for the AST1 and the AST3 in the nasal quadrant. ACD was shallower and LT was thicker in nanophthalmos, with WTW being larger in controls (*p* < 0.001). SFCT and RT were thicker in nanophthalmos compared to healthy individuals (*p* < 0.001). Conclusions: Significant anatomical differences are found in nanophthalmic eyes. They present a shallower ACD; thicker LT, OWT, choroid, and retina; and smaller WTW diameter—although no relevant differences in the AST were observed.

## 1. Introduction

Nanophthalmos is an uncommon eye condition characterized by abnormally small eyeballs with varying degrees of visual impairment [1,2,3]. The exact cause of nanophthalmos is not yet fully understood, but it is believed to be a genetic condition typically inherited with an autosomal recessive pattern. In these eyes, a shorter axial length (AXL) and a higher-than-normal lens-to-eye ratio are commonly found, but there are no definitive and unified criteria for the definition of nanophthalmos [1,2,3]. Nevertheless, nanophthalmos are not just smaller eyes because several morphologic peculiarities have been described in these eyes.

It is well-known that this abnormality can result in a higher risk of several eye conditions, such as high hyperopia, amblyopia, angle-closure glaucoma, retinal detachment, and cataracts. Indeed, Rajendrababu et al. described that legal blindness was present in 16.7% of their study sample [2].

Also, the increased interest in this entity is due to the frequent need for cataract and glaucoma surgery in these eyes. It is a challenge to calculate the intraocular lens (IOL) to be implanted in these eyes, and there is a well-known risk of refractive surprise [1,2,4]. Moreover, there are some peculiarities that different authors have proposed to be performed during cataract surgery, such as with prophylactic sclerotomy, in order to avoid intra and postoperative complications, which are frequent also after glaucoma surgery such as uveal effusion or postoperative shallow anterior chamber [4,5,6,7,8,9,10]. However, the mechanisms responsible for uveal effusion are not fully understood. A better understanding of the morphology of this entity could be helpful in reducing the risk of refractive surprise and uveal effusion.

Recently, the anatomical characteristics of nanophthalmic eyes have attracted much attention due to their clinical relevance. It has been postulated that nanophthalmos is characterized by a small eye where the anterior and posterior segments are reduced in size, with abnormally thickened sclera [1,2]. It is noteworthy that structures and measurements such as ocular wall or anterior scleral thickness (AST) have been postulated to play an important role in the pathogenesis of a variety of eye disorders, such as normotensive glaucoma, high myopia or central serous chorioretinopathy (CSCR) [11,12,13,14].

So far, the anterior segment dimensions of the eye have been investigated in vivo with different imaging techniques, mostly ultrasound biomicroscopy (UBM) and time-domain optical coherence tomography (OCT) [12]. Compared to these, newer swept-source (SS)-OCT technology offers improved resolution and tissue penetration for enhanced visualization and accurate measurements for both the anterior and posterior segments, including structures such as the anterior sclera or the choroid [15,16]. Thereby, some authors have suggested that measuring scleral thickness using AS-OCT could be useful in defining this condition [17].

However, no previous studies have analyzed the anterior and posterior segments of the eye in nanophthalmos compared to control eyes using SS-OCT, especially to figure out if a thickened anterior sclera could be a diagnostic criterion for this entity [18].

Hence, the present study was designed to assess the ocular morphology of the anterior and posterior segments in nanophthalmos compared to control eyes, especially the anterior scleral thickness, which is supposed to be involved in the pathogenesis of this condition and its complications.

## 2. Methods

### 2.1. Participants

A cross-sectional comparative study was carried out in two groups: 46 nanophthalmic eyes of 28 patients and 60 eyes of 30 controls paired by age and sex.

As nanophthalmos is really an uncommon condition, both eyes were included in the study. Out of a total of 5588 eyes measured in the biometrics database of Hospital Clínico San Carlos (IOLMaster^®^ 700, Carl Zeiss, Jena, Germany), only 66 eyes (0.0118%) had an AXL < 20.5 mm. However, some of them could not attend the clinical appointment to carry out the study protocol, and others did not meet the criteria to be included in the study. Thereby a muticentric approach was chosen. including also patients from other two hospitals (Hospital Universitario del Henares, Hospital Puerta de Hierro-Majadahonda).

Nanophthalmic eyes, forming the study group, were recruited from the biometrics database from the pre-surgical measurements of cataract surgery from October 2021 to May 2022 from these three hospitals. Healthy subjects, forming the control group, were recruited from their routine visits from October 2021 to May 2022 at general ophthalmology clinics of the Hospital Clínico San Carlos.

Both study and control group patients were invited to participate voluntarily in the study after the corresponding information and explanation. In order to participate, written informed consent was obtained from all participants. This study was performed with the approval of the Center’s Review Board (protocol code: 21/606.E) and in accordance with the Declaration of Helsinki.

Inclusion criteria in the study group were age ≥18 years, Caucasian race, and <20.5 mm of AXL as defined by different studies [1,3,4,19]. In the control group, the inclusion criteria were AXL between ≥20.5 mm and <26 mm, Caucasian race, and age ≥ 18 years.

Exclusion criteria were poor image quality or impossibility of acquiring the images due to physical or psychological reasons, any systemic disease (such as arterial hypertension or diabetes), presence of ocular abnormalities or malformations, concomitant ocular pathology (previously diagnosed or diagnosed during the examination) including corneal pathology (severe dry eye disease, keratitis or corneal dystrophy), ocular surface pathology (such as pterygium or pinguecula), retinal pathology (central serous chorioretinopathy, retinal detachment, diabetic retinopathy, hypertensive retinopathy or age-related macular degeneration), signs of intraocular inflammation, patients with anterior or posterior synechiae, intraocular pressure (IOP) > 21 mmHg, or previous ocular surgery (except cataract surgery).

### 2.2. Ophthalmological Examination

The participants underwent a medical history review, a comprehensive ophthalmological examination, and non-invasive imaging tests (biometry and OCT) on the same day. The ophthalmological examination consisted of visual acuity, slit-lamp biomicroscopy, Perkins tonometry, and funduscopy.

### 2.3. Ocular Biometry

All the candidates underwent AXL measurement by optical biometry before beginning the study if they did not already have a previous biometry from the calculation of the intraocular lens prior to cataract surgery.

Optical biometry was performed by the IOL Master^®^ 700 (Carl Zeiss, Jena, Germany), following the steps of the Intraocular Lens Calculation protocol and after ensuring that its calibration was correct. A previous adjustment was performed, including whether the patient was phakic (n = 33) or pseudophakic (n = 13). We explained to the participant that it was necessary to support the head, including the chin and forehead, and to look at the central light appearing through the optics. Only examinations classified by the software as “successful” were accepted.

The biometric parameters analyzed were the following:

- Axial Length (AXL) (mm): distance from the epithelium of the corneal apex to the inner limiting membrane (ILM) on the optical axis;
- Anterior Chamber Depth (ACD) (mm): distance from the corneal endothelium to the anterior lens capsule;
- Crystalline Lens Thickness (LT) (mm): distance from the anterior to the posterior pole of the lens ACD and LT were analyzed only in phakic patients in the group of nanophthalmos (n = 33);
- White-to-white (WTW) (mm): horizontal corneal diameter measured from limbus to limbus;
- Central Corneal Thickness (CCT) (µm): measured from the epithelium to the endothelium.

### 2.4. Optical Coherence Tomography

OCT was performed using two different devices: an SS-OCT, with a deeper penetration in tissues, and a spectral domain OCT (SD-OCT). All OCT scans were acquired by two well-trained examiners (ORQ and EMM). The anterior segment images were acquired prior to pupil dilation, and those of the posterior pole were obtained after pupil dilation.

The SS-OCT employed was Plex Elite 9000 (Carl Zeiss Meditec, Dublin, CA, USA). This device uses a central wavelength between 1040 nm and 1060 nm with an axial resolution of 6.3 μm, a scan depth of 3 mm, and a scanning speed of 100,000 A-scans per second. A prototype 10-diopter anterior segment lens provided by the device was incorporated to obtain cross-sectional images of the anterior sclera using the raster “HD line” anterior segment capture mode with a scan field of 6 mm. Posterior pole images corresponding to the measurement of subfoveal choroidal thickness (SFCT) and central retinal thickness (CRT) were obtained with “HD Spotlight” mode.

The SD-OCT employed was Spectralis (Spectralis, Heidelberg Engineering Inc., Heidelberg, Germany). This system takes 40,000 axial scans per second and has a 7 μm axial resolution. Image acquisition was performed by incorporating the Anterior Segment Module lens provided by Spectralis and selecting “Sclera” mode with the raster “Large” to obtain cross-sectional horizontal images of 6 mm.

For the examination of the anterior scleral wall at 3- and 9-o’clock positions, the participants were asked to adopt a maximal temporal or nasal gaze during scanning. In order to obtain a stable fixation during the OCT examination, an external fixation light was used, with the patient’s chin and forehead firmly fixed to the device to minimize head or eye position changes during the examination. To obtain the images of the posterior pole, participants were asked to look at the light coming from the OCT optics to keep the eyes in the primary position. Only images of sufficient quality were accepted. All OCT scans were performed in the same room under mesopic lighting conditions.

A secondary objective of the present study was to assess the correlation of the SS-OCT and SD-OCT measurements of the AST.

### 2.5. OCT Measurements

**Figure 1:**
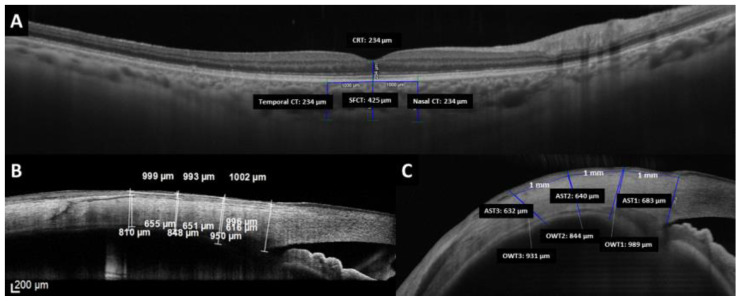
Measurements made by optical coherence tomography of the (**A**) central retinal thickness (CRT), subfoveal choroidal thickness (SFCT), and choroidal thickness at 1 mm nasal and 1 mm temporal from the fovea. (**B**,**C**) Measurements of the ocular wall thickness and anterior scleral thickness by the SD-OCT device and SS-OCT device, respectively.

Ocular wall thickness (OWT) refers to the measurement of the complete anterior ocular wall, including conjunctiva, sclera, and ciliary body or choroid (Figure 1). For the AST measurement in the OCT images, the external limit of the sclera can be identified by the deep episcleral vascular plexus, which manifests as a thin hyporeflective region below the conjunctiva-Tenon capsule. The inner boundary is a sharply demarcated line between the hyper-reflective scleral tissue and the hyporeflective ciliary body tissue. OWT and AST were manually measured by the same investigator (ORQ) in a masked fashion in the temporal and nasal quadrants at 1 (OWT1 and AST1), 2 (OWT2 and AST2), and 3 mm (OWT3 and AST3) from the scleral spur.

Also, the SFCT, choroidal thickness (CT) at 1 mm nasal and temporal to the fovea, and CRT were measured using the PlexElite device (Figure 1). The reproducibility of the intra and interobserver choroid measurements was studied. To determine intraobserver reproducibility, one expert examiner also took measurements on the same images 2 months after the first measurements. For interobserver reproducibility, measurements were independently made on the images obtained in the initial examination by two expert observers.

### 2.6. Statistical Analysis

Statistical analysis was conducted using the software package SPSS^®^ (Statistical Package for Social Sciences, v21.0; SPSS Inc., Chicago, IL, USA). Mean and standard deviation are used to depict quantitative data, while qualitative data are expressed as frequency distributions. The Kolmogorov–Smirnov test confirmed the normal distribution of data. T-test was employed to evaluate differences between groups. The correlation of the AST measurements between the SS-OCT and SD-OCT was calculated by the Pearson correlation coefficient. Also, the correlation between AXL and the different ocular parameters assessed in the nanophthalmic eyes was calculated. In the reproducibility analysis, for each measurement, the intraclass correlation coefficient (ICC; two-way mixed effects, absolute agreement, single measurement) was calculated for the two consecutive scans. Lastly, Bland–Altman plots were calculated to analyze the agreement between the choroid measurements. Statistical significance was considered when *p* < 0.05.

## 3. Results

Forty-six nanophthalmic eyes of 28 patients and 60 healthy eyes of 30 control subjects were included. There was no difference in either the mean age or in the gender between groups (*p* > 0.05).

**Figure 2:**
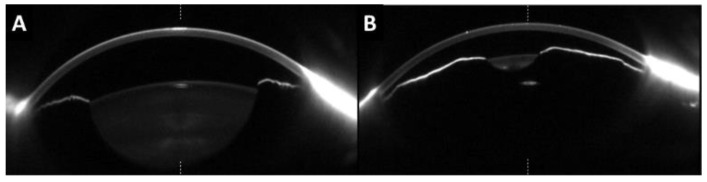
Anterior segment exploration of two different examples of nanophthalmic eyes by Scheimpflug camera. (**A**) Large lens thickness; (**B**) Shallow anterior chamber depth.

Mean AXL was 19.3 ± 1.5 mm in the nanophthalmos group and 23.9 ± 1.1 mm in the control group (*p* < 0.001) (Table 1). ACD was shallower in nanophthalmos (3.06 ± 0.91 vs. 3.46 ± 0.48 mm, *p* < 0.001), LT was large in nanophthalmos (4.44 ± 0.58 vs. 4.12 ± 0.48 mm, *p* < 0.001) (Figure 2), and WTW was larger in controls (12.1 ± 0.40 vs. 11.6 ± 0.48 mm, *p* < 0.001).

On the other hand, no differences were observed in CCT (549.0 ± 35.5 vs. 540.6 ± 38.4 µm for nanophthalmos and controls, respectively, *p* = 0.253).

Regarding the choroid, both the SFCT (447 ± 125 vs. 307 ± 90, *p* < 0.001) and the nasal and temporal CT (440 ± 131 vs. 273 ± 87 and 424 ± 120 vs. 292 ± 82, *p* < 0.001) were thicker in the nanophthalmic eyes compared to controls (*p* < 0.001). Also, the CRT was larger in the nanophthalmic eyes than in controls (292 ± 80 vs. 252 ± 51, *p* < 0.001).

In the assessment of the anterior wall dimensions, the OWT was thicker in all measurement points (at 1, 2, and 3 mm from the scleral spur) and in both quadrants in nanophthalmic eyes (*p* < 0.001) (Table 2).

**Figure 3:**
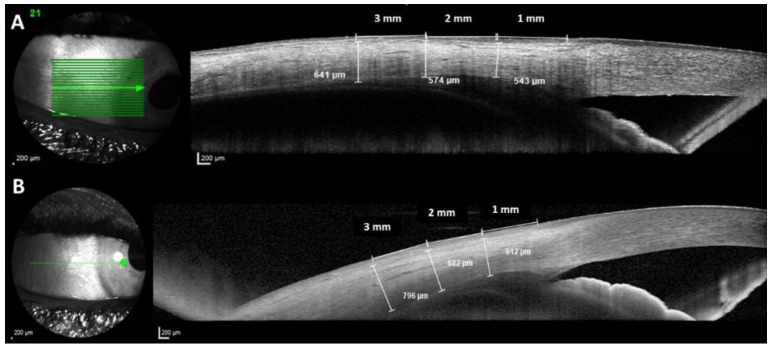
Optical coherence tomography of two examples of nanophthalmic eyes with an especially short axial length (AXL) ((**A**): 18.5 mm of AXL; (**B**): 15.94 mm of AXL) showing the anterior scleral thickness (AST) measurements at 1, 2, and 3 mm from the limbus.

There were no differences in the AST in almost all measurements performed between groups (*p* ≥ 0.194), except for the nasal AST1 measured by SS-OCT, temporal AST1, and nasal AST3 measured by SD-OCT (*p* ≤ 0.039) (Figure 3).

Comparing the AST measurements between the eyes with AXL < 20 mm (n = 24) to the ones from ≥20 to <20.5 mm (n = 22), no differences emerged in any of the parameters studied (*p* ≥ 0.345) (Figure 3).

There was a weak correlation between the AST measurements made between both OCT devices (SS-OCT and SD-OCT), being R = 0.343, 0.262, and 0.294 for the nasal measurements at 1, 2, and 3 mm, respectively (all *p* < 0.05), and R = 0.163, 0.173 and 0.265 for the temporal measurements at 1, 2 and 3 mm, respectively.

A mild correlation was observed between the AXL and the AST2 and 3 (R = −0.322 and −0.478, *p* ≤ 0.038) and between AXL and OWT2 and OWT3 (R = −0.432 and −0.540; *p* ≤ 0.004) both in the temporal quadrant. Also, AXL and LT were correlated (R = −0.366, *p* = 0.008) (Table 3).

However, no correlation was observed between the AXL and ACD, CCT, WTW, OWT, and AST in the nasal quadrants (all *p* < 0.135).

**Figure 4:**
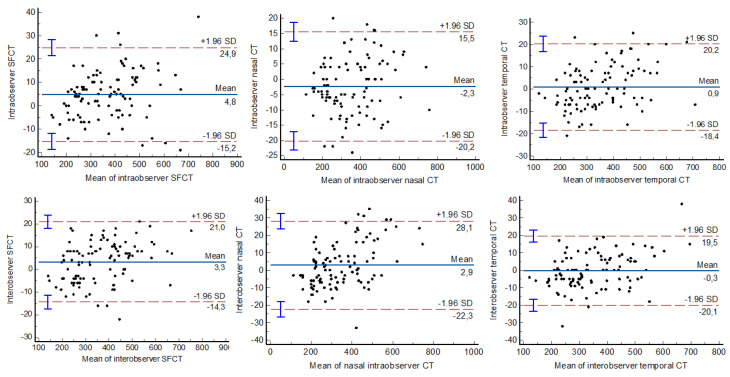
Bland–Altman plots showing the agreement between the choroid measurements.

The reproducibility of the choroid measurements was excellent (ICC ≥ 0.991) in nanophthalmos and in the control group, both for intraobserver and interobserver measurements (Table 4). Bland–Altman plots for the choroid measurements showed excellent agreement (Figure 4).

## 4. Discussion

Nanophthalmos has recently gained much interest due to its clinical and surgical implications and comorbidities [1,2,3]. Diagnostically, AXL below 20.5 mm and retinochoroidal scleral thickness (RCS) > 1.7 mm measured by B-scan with no associated ocular malformations are the main criteria for nanophthalmos up to date as proposed by different authors [1,2,3]. There is currently a lack of recognized diagnostic criteria for nanophthalmos, and other anatomical parameters have been proposed to be included in its definition, such as thickened sclera. A thickened sclera supposedly plays a central role in uveal effusion, one of the more common and feared complications of glaucoma and cataract surgery in these eyes. Nevertheless, despite being accepted by most ophthalmologists, the evidence that supports this theory is weak. However, the presence of a thickened sclera in nanophthalmos has been described only in isolated case reports, usually measured using inaccurate methods like echography, but has not been described either in a large study or using OCT. Moreover, no cut-off values have been proposed for AST as a diagnostic criterion in nanophthalmos.

It is noteworthy that in the present study, the OWT was thicker in all measurement points (at 1, 2, and 3 mm from the scleral spur) and in both quadrants in nanophthalmos eyes (*p* < 0.001). By contrast, there were no differences in the AST between groups in almost all measurements performed (*p* ≥ 0.194), except for the nasal AST1 measured by SS-OCT, temporal AST1, and nasal AST3 measured by SD-OCT (*p* ≤ 0.039). Since the OWT measurements include the AST dimensions, the possible explanation for the difference in OWT measurement could be a thicker anterior choroid or ciliary body in the group of nanophthalmos. Despite being significant from a statistical point of view, we hypothesize that these differences in the range of 10% are probably non-significant from a biological point of view. In addition, Figure 3 shows the AST measurements in nanophthalmic eyes with especially short AXL. In this regard, Kaewsangthong et al. measured the AST in a nanophthalmic patient using a UBM to avoid the influence of uveal leakage and detachment on the RCS measurement results, showing that the scleral thickness at the limbus of this patient was 1.26 mm [20], while the scleral thickness at the limbus of a normal eyeball was 0.53 ± 0.14 mm [21]. Interestingly, Lu et al., using anterior segment SS-OCT in nanophthalmic eyes, found that the morphological features of the aqueous humor pathway, including Schlemm’s canal and trabecular meshwork dimensions, are significantly smaller than those of normal eyes [8]. Therefore, probably the angle closure and aqueous humor pathway changes could be responsible for glaucoma rather than a thick anterior sclera in nanophtalmic eyes.

Tailor et al. believed that the scleral thickening and disordered structure are the causes of nanophthalmos and defended that scleral thickening should be a necessary condition for nanophthalmos diagnosis [7]. Different authors have previously confirmed by histology that perifibrillar aggregates, similar to proteoglycans, were prominent in the nanophthalmic sclera. Also, the sclera was thicker than normal, and the bundles of collagen fibrils were less ordered [22,23].

Multiple theories are based on the fact that there is a restriction in eyeball growth, subsequently producing the characteristic associated complications of nanophthalmos. It has been previously described that nanophthalmic eyes are prone to develop uveal effusion either from the thickening of the sclera or from the reduced scleral permeability [4]. He et al. have also described that the pathogenesis of fluid misdirection syndrome and uveal effusion syndrome in nanophthalmos could be related to the scleral thickening and abnormal deposition of glycosaminoglycans (with higher hydrophilicity), which will increase choroidal osmotic pressure, hinder trans scleral protein transport and inhibit the drainage of vortex veins causing choroidal effusion [5]. In addition, Calhoun proposed that a posterior thick scleral wall compresses the vortex veins at their exit from the ocular globe, causing choroidal congestion, which is probably responsible for serous choroidal detachment after glaucoma surgery in these eyes [24].

**Figure 5:**
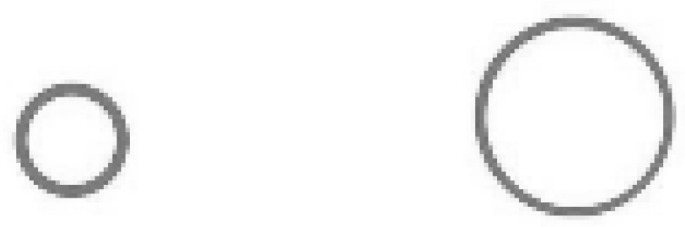
The Ebbinghaus illusion. A scleral layer of the same thickness will appear thicker in a smaller eye.

Given the results found in the present study, we believe that the increase in the thickness of the OWT with an associated sclera with altered properties could probably be responsible for complications such as uveal effusion, although future studies are required to confirm this hypothesis. Our findings clash with the traditional idea that nanophthalmic eyes have thicker sclera. We propose two theories to explain this paradox. Current technology does not allow detailed mapping of the whole sclera. Therefore, it is possible that the anterior nasal and temporal regions are not representative of the whole layer. Nevertheless, with the sclera being a continuous layer, this theory is counterintuitive. The other possibility is that ocular surgeons have traditionally made incorrect estimations of the scleral thickness. Ocular volume can bias the perception of scleral thickness by a mechanism related to the so-called Ebbinghaus illusion, which consists of the fact that the perceived dimensions of an object are influenced by the dimensions of close objects (Figure 5). Therefore, an ocular wall of the same thickness will appear thicker in a smaller eye than in a bigger one [25].

In the present study, even when a weak correlation between the AST measurements with both OCT technologies (SS-OCT and SD-OCT) was found, tight differences between nanophthalmos and controls were observed in all cases.

Regarding the complete anatomical assessment performed in the present study, we found that there are significant anatomical differences in several structures analyzed in nanophthalmic eyes compared to controls. As for the classic parameters of the anterior segment, ACD was shallower, and LT was thicker in nanophthalmos, with WTW being larger in controls, whereas no differences were observed in CCT. This is congruent with the previous descriptions and definitions made by different authors.

Rajendrababu et al. described that the mean AXL (17.64 ± 1.74 mm) was inversely correlated to the mean RCS thickness (R = −0.28, *p* < 0.001) measured by B-scan [2]. This could be relevant to decide whether or not to perform a prophylactic sclerotomy. In the present study, a similar inverse mild correlation (ranging from R = −0.322 and −0.540) was also observed between the AXL and the AST and OWT in the temporal quadrant. However, no correlation was observed for these parameters in the nasal sector, and neither between AXL and ACD, CCT, or WTW.

In relation to the higher-than-normal lens-to-eye ratio, we found an LT in nanophthalmos similar to the value offered by Rajendrababu et al. (4.44 ± 0.58 mm vs. 4.27 ± 0.70 mm) [2].

Rajendrababu et al., in phakic nanophthalmos, found a mean ACD of 2.34 ± 0.59 mm. In agreement, in the present study, ACD was shallower in nanophthalmos (3.06 ± 0.91 vs. 3.46 ± 0.48 mm, *p* < 0.001) than in controls [2]. It should be highlighted that differences in the measurements between different studies could be related to the degree or severity of the nanophthalmos.

A corneal diameter < 11 mm has also been proposed in two different studies as diagnostic criteria for nanophthalmos [1]. In agreement, in the present study, the WTW was shorter in nanophthalmos than in controls (11.6 ± 0.48 mm vs. 12.1 ± 0.40 mm, *p* < 0.001). Relhan et al. differentiated nanophthalmos and posterior microphthalmos considering the horizontal corneal diameter in their study published in 2015 [26]. Corneal diameter ≥11 mm was classified as PM, whereas ≤11 mm was considered as nanophthalmos. However, in our study, only four eyes in the group of nanophthalmos (out of 46) had a WTW < 11 mm.

Finally, about the posterior segment parameters analyzed, the SFCT (447 ± 125 vs. 307 ± 90 µm, *p* < 0.001) and the nasal and temporal CT (440 ± 131 vs. 273 ± 87 and 424 ± 120 vs. 292 ± 82, *p* < 0.001) were thicker in the nanophthalmic eyes compared to controls (*p* < 0.001). Similarly, Demircan et al. first described the SFCT of nanophthalmic patients, observing a mean of 551 ± 87 μm in the nanophthalmos group while it was 330 ± 46 μm in the control group [27]. The higher CT values in their study could be related to the fact that they have included nanophthalmos with shorter AXL. Aksoy et al. also presented a relative increase in the CT on the nasal vs. the temporal side, similar to the findings observed here [28]. It is well-known that the CT of healthy adults is thickest at the fovea, followed by the temporal side being the thinnest part on the nasal side [29]. In contrast, Kaneko et al. described a CT in nanophthalmos thickest in the subfoveal location, followed by the nasal side, with the temporal side being the thinnest part of the choroid [30]. Additionally, Wu et al. found that an increased RCS thickness (mean 2.41 mm) confirmed the diagnosis of nanophthalmos by echography [6]. Other authors have also considered this increased posterior scleral thickening or combined RCS thickening as an additional diagnostic criterion [6,9,10].

Yang et al. have described that during normal eye development, the choroid extends from the optic disc to the temporal side, while the nasal side becomes thinner [1]. Then, these authors hypothesized that as the choroid and sclera have a common embryological origin, the choroid of nanophthalmic patients does not develop properly, so it cannot fully stretch, with the result being a relative thickening of the choroid near the optic disc on the nasal side.

Also, in our population, the CRT was larger in the nanophthalmic eyes than in controls (292 ± 80 vs. 252 ± 51, *p* < 0.001). In agreement, Demircan et al. report that the average CRT in nanophthalmic patients (331.9 ± 78.9 μm) was significantly higher than in the control group (268.9 ± 24.3 μm) [27].

The main clinical relevance of nanophthalmos is that, as Rajendrababu et al. have previously described, at presentation, 38.2% had moderate visual impairment, 19.4% had severe visual impairment, and 16.7% of patients were legally blind [2]. The main causes of blindness included glaucomatous optic atrophy (54.2%), retinitis pigmentosa (20.8%), and choroidal effusion (12.5%). Also, high hyperopia with amblyopia is a common cause of impaired vision in these patients. The management of its typical high hyperopia with amblyopia, angle-closure glaucoma, or cataracts and its complications are still challenges for many treating physicians worldwide.

The present study has some limitations. Firstly, we only assessed the horizontal quadrants and not the vertical quadrants for the OWT and AST measurements. Secondly, the spherical error was not registered, but the AXL, which we consider a more appropriate parameter to define these eyes, was measured.

Notwithstanding, this is the first study to use SS-OCT to assess several parameters of the eyeball in nanophthalmos in a case–control study with a relatively large sample size of these patients taking into account its incidence. Also, this is the first time, to the authors’ knowledge, that AST dimensions in the most anterior portion of the sclera in nanophthalmos eyes were studied by SS-OCT. Strikingly, no differences were observed between groups, so we are not able to propose this parameter in the definition of nanophthalmos.

It should be highlighted that the use of inconsistent criteria for the clinical diagnosis and classification of severity of nanophthalmos in different studies leads to differences in research results. Therefore, we consider it a necessity to reach a consensus to unify the main criteria that define not only nanophthalmos but also its severity. Future studies with a larger population and with measurements at different scleral sites should be performed to confirm these results. In addition, not only the thickness but also the rigidity and other properties of the sclera in nanophthalmic eyes should be analyzed.

In conclusion, there are significant anatomical differences in nanophthalmic eyes, having a shallower ACD, a thicker OWT, a smaller WTW diameter, and a thicker choroid and retina, observing no relevant differences in the AST.

## Figures and Tables

**Table 1:** Measurements of different ocular parameters comparing the nanophthalmos and the control group. Mean ± standard deviation (range).

Variable	Nanophthalmos	Control Group	*p*-Value
AXL (mm)	19.3 ± 1.5 (15.48–20.47)	23.9 ± 1.1 (21.84–26.97)	<0.001
CCT (µm)	549.0 ± 35.5 (479–625)	540.6 ± 38.4 (412–644)	0.253
WTW (mm)	11.6 ± 0.48 (10.60–13.02)	12.1 ± 0.40 (11.40–13.20)	<0.001
ACD (mm)	3.06 ± 0.91 (1.97–5.06)	3.46 ± 0.48 (2.83–4.66)	<0.001
LT (mm)	4.44 ± 0.58 (3.26–5.47)	4.12 ± 0.48 (3.25–5.42)	<0.001
SFCT (µm)	447 ± 125 (192–761)	307 ± 90 (139–545)	<0.001
Nasal 1 mm CT (µm)	440 ± 131 (177–755)	273 ± 87 (111–498)	<0.001
Temporal 1 mm CT (µm)	424 ± 120 (182–704)	292 ± 82 (119–500)	<0.001
CRT (µm)	292 ± 80 (205–535)	252 ± 51 (186–545)	<0.001

AXL: axial length; CCT: central corneal thickness; WTW: white-to-white; ACD: anterior chamber depth; LT: lens thickness; SFCT: subfoveal choroidal thickness; CT: choroidal thickness; CRT: central retinal thickness.

**Table 2:** Dimensions of the ocular wall structures by optical coherence tomography in nanophthalmos and control eye group. Mean ± standard deviation (range).

Variable	Nanophthalmos	Control Group	*p*-Value
Nasal OWT1 (µm)	1185 ± 185 (881–1761)	1064 ± 120 (651–1315)	<0.001 *
Nasal OWT2 (µm)	1066 ± 208 (747–1757)	948 ± 96 (715–1181)	<0.001 *
Nasal OWT3 (µm)	1020 ± 222 (554–1579)	923 ± 120 (562–1218)	0.005 *
Temporal OWT1 (µm)	1157 ± 166 (776–1594)	1049 ± 116 (615–1303)	<0.001 *
Temporal OWT2 (µm)	996 ± 190 (729–1811)	916 ± 104 (635–1222)	0.007 *
Temporal OWT3 (µm)	981 ± 253 (560–1823)	874 ± 121 (554–1163)	0.005 *
Nasal AST1 (µm) SS-OCT	742 ± 91 (590–983)	687 ± 98 (505–1180)	0.004 *
Nasal AST2 (µm) SS-OCT	769 ± 137 (547–1271)	740 ± 94 (565–1060)	0.199
Nasal AST3 (µm) SS-OCT	755 ± 146 (400–1207)	767 ± 120 (458–990)	0.654
Temporal AST1 (µm) SS-OCT	693 ± 101 (523–951)	670 ± 100 (535–1229)	0.249
Temporal AST2 (µm) SS-OCT	695 ± 129 (400–1017)	704 ± 89 (516–923)	0.688
Temporal AST3 (µm) SS-OCT	705 ± 188 (358–1333)	691 ± 118 (402–943)	0.656
Nasal AST1 (µm) SD-OCT	705 ± 102 (547–1003)	682 ± 99 (540–1002)	0.238
Nasal AST2 (µm) SD-OCT	732 ± 106 (553–1008)	707 ± 84 (547–998)	0.194
Nasal AST3 (µm) SD-OCT	759 ± 120 (566–1042)	719 ± 69 (562–902)	0.039 *
Temporal AST1 (µm) SD-OCT	654 ± 79 (527–841)	691 ± 99 (518–1154)	0.038 *
Temporal AST2 (µm) SD-OCT	658 ± 83 (497–850)	666 ± 87 (519–991)	0.628
Temporal AST3 (µm) SD-OCT	691 ± 96 (550–956)	691 ± 74 (577–955)	0.994

OWT: ocular wall thickness; AST: anterior scleral thickness; SS-OCT: swept-source optical coherence tomography; SD-OCT: spectral domain optical coherence tomography; *: statistically significant.

**Table 3:** Correlation between axial length (AXL) and different ocular structure dimensions in the nanophthalmic eyes.

Variable	Correlation with AXL (*p*-Value)
ACD	R = 0.029 (*p* = 0.847)
LT	R = −0.366 (*p* = 0.008)
WTW	R = 0.150 (*p* = 0.319)
CCT	R = −0.097 (*p* = 0.517)
SFCT	R = −0.216 (*p* = 0.113)
Nasal 1 mm CT	R = −0.242 (*p* = 0.075)
Temporal 1 mm CT	R = −0.112 (*p* = 0.416)
Retinal thickness	R = 0.079 (*p* = 0.567)
Nasal OWT1	R = 0.103 (*p* = 0.520)
Nasal OWT2	R = −0.036 (*p* = 0.826)
Nasal OWT3	R = −0.054 (*p* = 0.745)
Temporal OWT1	R = −0.045 (*p* = 0.779)
Temporal OWT2	R = −0.432 (*p* = 0.004)
Temporal OWT3	R = −0.540 (*p* = 0.001)
Nasal AST1 SS-OCT	R = −0.018 (*p* = 0.911)
Nasal AST2 SS-OCT	R = −0.238 (*p* = 0.135)
Nasal AST3 SS-OCT	R = −0.076 (*p* = 0.647)
Temporal AST1 SS-OCT	R = −0.090 (*p* = 0.572)
Temporal AST2 SS-OCT	R = −0.322 (*p* = 0.038)
Temporal AST3 SS-OCT	R = −0.478 (*p* = 0.001)
Nasal AST1 SD-OCT	R = 0.230 (*p* = 0.087)
Nasal AST2 SD-OCT	R = −0.089 (*p* = 0.512)
Nasal AST3 SD-OCT	R = 0.147 (*p* = 0.280)
Temporal AST1 SD-OCT	R = −0.181 (*p* = 0.223)
Temporal AST2 SD-OCT	R = −0.357 (*p* = 0.015)
Temporal AST3 SD-OCT	R = −0.272 (*p* = 0.075)

AXL: axial length; CCT: central corneal thickness; WTW: white-to-white; ACD: anterior chamber depth; LT: lens thickness; SFCT: subfoveal choroidal thickness; CT: choroidal thickness; CRT: central retinal thickness; OWT: ocular wall thickness; AST: anterior scleral thickness; SS-OCT: swept-source optical coherence tomography; SD-OCT: spectral domain optical coherence tomography.

**Table 4:** Reproducibility of choroid measurements by optical coherence tomography in nanophthalmos and control eye group. Mean ± standard deviation (range).

Nanophthalmos	SFCT (µm)	Nasal 1 mm CT (µm)	Temporal 1 mm CT (µm)
Observer 1: First measurement	447 ± 125 (192–761)	440 ± 131 (177–755)	424 ± 120 (182–704)
Observer 1: Second measurement	442 ± 123 (191–723)	442 ± 130 (181–765)	419 ± 117 (189–711)
Observer 2	443 ± 123 (201–744)	434 ± 125 (195–740)	423 ± 115 (189–689)
Intraobserver ICC	0.995 (0.989–0.998)	0.997 (0.995–0.999)	0.995 (0.989–0.998)
Interobserver ICC	0.996 (0.991–0.998)	0.991 (0.978–0.996)	0.996 (0.993–0.998)
**Control Group**	**SFCT (µm)**	**Nasal 1 mm CT (µm)**	**Temporal 1 mm CT (µm)**
Observer 1: First measurement	307 ± 90 (139–545)	273 ± 87 (111–498)	292 ± 82 (119–500)
Observer 1: Second measurement	303 ± 87 (143–559)	276 ± 85 (116–505)	295 ± 80 (121–491)
Observer 2	305 ± 88 (142–537)	274 ± 85 (114–503)	293 ± 79 (123–495)
Intraobserver ICC	0.993 (0.985–0.996)	0.994 (0.990–0.997)	0.994 (0.990–0.997)
Interobserver ICC	0.995 (0.992–0.997)	0.994 (0.990–0.997)	0.993 (0.988–0.996)

ICC = intraclass correlation coefficient (95% confidence interval); SFCT: subfoveal choroidal thickness; CT: choroidal thickness.

## Data Availability

Data used to support the findings presented in this study are available on request from the corresponding author.
